# HOUSEHOLDS AND LIVING ARRANGEMENTS OF OLDER PERSONS AROUND THE WORLD

**DOI:** 10.1093/geroni/igz038.2967

**Published:** 2019-11-08

**Authors:** Yumiko Kamiya, Yumiko Kamiya, Sara Hertog

**Affiliations:** United Nations, New York, New York, United States

## Abstract

The household living arrangements of older persons – whether living alone, with a spouse or partner, with their children or in multi-generational households – can be an important factor associated with their health, economic status and overall well-being. Understanding the patterns and trends in older persons’ living arrangements is thus relevant for global efforts to achieve the sustainable development goals, in particular those targeting poverty, hunger and health. The United Nations Database on the Households and Living Arrangements of Older Persons 2018 presents evidence drawn from 672 unique data sources, including census and survey microdata samples archived at IPUMS-International and household rosters from Demographic and Health Surveys, among other sources. The resulting dataset describes older persons’ households across 147 countries or areas, representing approximately 97 per cent of persons aged 60 or over globally.

